# Host Immune Response Mechanisms Against Herpes Simplex Virus Type 2 Infection

**DOI:** 10.3390/pathogens15030319

**Published:** 2026-03-16

**Authors:** Yongming Mei, Hong Teng, Jianbin Wang

**Affiliations:** The Public Experimental Center of Medicine, Affiliated Hospital of Zunyi Medical University, 149 Dalian Road, Huichuan District, Zunyi 563003, China

**Keywords:** herpes simplex virus type 2, latent infection, innate immunity, adaptive immunity, immune evasion

## Abstract

Herpes simplex virus type 2 (HSV-2) is the primary pathogen responsible for genital herpes. Predominantly transmitted via sexual contact, HSV-2 not only poses significant physical and psychological burdens on infected individuals but also substantially elevates the risk of HIV acquisition and represents a potentially fatal threat to newborns. Following primary infection, HSV-2 establishes lifelong latent infection within the sacral ganglia. Currently, there are no vaccines or therapeutics capable of eradicating this latent virus reservoir or effectively preventing initial infection. The core impediment to developing such interventions lies in the incomplete elucidation of the protective immune mechanisms against HSV-2 and its precise molecular pathogenesis. The host immune response against HSV-2 hinges critically on the coordinated interplay between innate and adaptive immunity. The innate immune system, serving as the first line of defense, acts to curtail early viral replication and initiate adaptive responses. This is achieved through mechanisms, including the genital mucosal barrier, activation of Toll-like receptors (TLRs), the cGAS-STING signaling pathway, interferon (IFN)-mediated antiviral effector functions, and activation of innate immune cells such as natural killer (NK) cells and dendritic cells (DCs). Crucially, however, HSV-2 counteracts these host defenses by expressing immune modulatory proteins (e.g., ICP0, ICP27, ICP35) that target key host antiviral signaling pathways, thereby affecting immune evasion. Within the adaptive immune response, neutralizing antibodies generated by the humoral immunity can provide localized protection at mucosal sites, but their protective efficacy is limited due to sophisticated viral immune evasion mechanisms. Cellular immunity, particularly mediated by CD4+ T cells, constitutes the core mechanism for viral clearance and suppression of recurrent outbreaks. Notably, tissue-resident memory T cells (TRMs) play a pivotal role in controlling the reactivation of latent HSV-2 within the ganglia. This review integrates current research advances to delineate the innate and adaptive immune mechanisms engaged during HSV-2 infection from the perspective of the dynamic host–virus interplay, with an ultimate aim to provide a theoretical foundation informing the rational development of preventive vaccines and therapeutic agents against HSV-2.

## 1. Introduction

Herpes simplex virus type 2 (HSV-2), classified within the subfamily *Alphaherpesvirinae* of the family *Herpesviridae*, is an enveloped, linear double-stranded DNA virus primarily transmitted through sexual contact. It is the principal causative agent of genital herpes [1]. HSV-2 infection elevates the risk of HIV acquisition and transmission by 3–4-fold [2,3] and poses a potentially fatal threat to neonates delivered through an infected birth canal. Globally, approximately 14,000 new cases of neonatal HSV-2 infection occur annually, carrying a high mortality rate in untreated neonates [4,5]. Following primary infection, HSV-2 can establish a lifelong latent infection within the sacral ganglia [6]. During episodes of transient immune suppression triggered by factors such as fever, cold exposure, emotional stress, co-infection with other pathogens, or compromised cellular immunity, the latent virus periodically reactivates. Reactivated virus travels anterograde along sensory nerve axons to the peripheral epithelial cells, where it replicates. This replication leads to asymptomatic low-titer viral shedding or, less commonly, prolonged high-titer shedding, ultimately resulting in recurrent ulcerative lesions [7]. Currently, specific therapeutic drugs for HSV-2 can only control clinical symptoms but cannot eradicate the latent virus or prevent recurrence, imposing a persistent burden on human health.

The host immune response to HSV-2 infection comprises innate and adaptive immunity. As the body’s initial defense, innate immunity provides a rapid, non-specific response against pathogen invasion, crucially controlling primary infection and inhibiting viral replication [8]; subsequently, adaptive immunity is activated to synergistically regulate the course of infection with innate immunity [9,10]. However, the mechanisms of protective immunity against HSV-2 infection remain incompletely understood, and effective preventive or therapeutic vaccines for controlling infection are still under development [11]. In recent years, breakthroughs have been made in fields such as cGAS-STING signaling pathway regulation, broad-spectrum neutralizing antibody development, and mRNA vaccine technology, providing new directions for deciphering immune response mechanisms and developing novel intervention strategies. Therefore, in-depth investigation of the host immune response following HSV-2 infection has become a central focus for advancing vaccine development and deepening the understanding of pathogenic mechanisms and antiviral immunity. From the perspective of bidirectional host–virus interaction, this article reviews the research progress on innate and adaptive immune responses to HSV-2 infection, clarifies their critical roles in host protection, and provides theoretical support for the development of related vaccines and therapeutic agents. It should be noted that although some of the mechanistic studies originated from HSV-1 research, given the structural similarity between HSV-1 and HSV-2, the findings may also be applicable to HSV-2.

## 2. Innate Immune Response to HSV-2 Infection

The core functions of the human innate immune system in defending against HSV-2 infection include suppressing viral replication to block infection spread and activating adaptive immune responses. The genital tract mucosa serves as the primary site for HSV-2 initial infection. Its stratified squamous epithelial cells not only form a physical barrier but also secrete antimicrobial factors such as β-defensins, secretory leukocyte protease inhibitor (SLPI), and lysozyme, establishing an intrinsic antimicrobial microenvironment [12]. As key pattern recognition receptors (PRRs), Toll-like receptors (TLRs) on cellular surfaces recognize HSV-2 pathogen-associated molecular patterns (PAMPs), thereby functioning as the essential bridge initiating innate immunity and linking it to adaptive immunity. Inflammatory responses and cytokines produced at the initial infection site can recruit various innate immune cells such as inflammatory monocytes, natural killer (NK) cells, dendritic cells (DCs), and plasmacytoid dendritic cells (pDCs) to collaboratively participate in the antiviral response [13]. Conversely, through long-term co-evolution with host innate immunity, HSV-2 has evolved multiple immune evasion strategies. It encodes functional proteins, such as immediate–early proteins and envelope glycoproteins, that target host antiviral signaling pathways to facilitate viral survival and proliferation [14].

### 2.1. The Central Roles of TLRs and the cGAS-STING Signaling Pathway in Innate Immunity Against HSV-2

TLRs are essential PRRs involved in immune system development and function exertion, consisting of an extracellular domain, a cytoplasmic domain, and a transmembrane domain. The extracellular domain is rich in leucine-rich repeats (LRRs) and is responsible for recognizing PAMPs. TLRs activate the transcription of immune response genes by inducing the secretion of cytokines such as interleukins (ILs) and interferons (IFNs), thereby achieving host protection [15]. Studies have confirmed that TLR-2, TLR-3, TLR-4, and TLR-9 can specifically recognize components of HSV-2 such as glycoproteins (gB, gH, gK, gL) and US2 protein, participating in the process of viral infection and reactivation [16]. During HSV-2 invasion, TLR-2 on the surface of DCs can form heterodimers with TLR-6 or TLR-1 to recognize viral glycoproteins gH and gL [17]; TLR-4 recognizes viral short hairpin DNA on the cell surface [18], and TLR-9 is activated by viral CpG-rich DNA [19]. The activation of these TLRs initiates MyD88-dependent signal cascades: MyD88 forms a complex with IL-1 receptor-associated kinase 1 (IRAK1) and tumor necrosis factor receptor-associated factor 6 (TRAF6), recruiting transforming growth factor-β-activated protein kinase 1 binding protein 2 (TAB2) and transforming growth factor-β-activated kinase 1 (TAK1); TRAF6 then induces the phosphorylation of TAK1, leading to activation of the inhibitor of nuclear factor κB kinase (IKK) complex, which results in the phosphorylation and degradation of the inhibitor of nuclear factor κB (IκB), allowing NF-κB to translocate into the nucleus. Concurrently, TAK1 activates the mitogen-activated protein kinase (MAPKs) pathway, triggering the nuclear translocation of activated protein 1 (AP-1). Ultimately, the activation of NF-κB and AP-1 promotes immune cells to secrete IL-15, TNF-α, and IFN-β to resist viral infection [16]. Furthermore, TLR-2 and TLR-9 can cooperate to synergistically enhance the innate immune response against HSV-2 [20].

**Figure 1:**
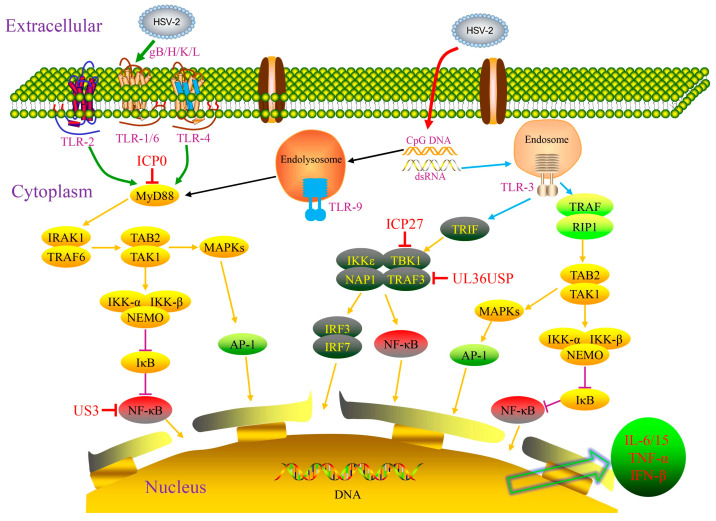
TLR-mediated signaling pathways in innate immunity against HSV-2. TLRs, located on the plasma membrane and within endosomes, sense viral ligands such as dsRNA, dsDNA, and glycoproteins. TLR2, TLR4, and TLR9 recruit MyD88. MyD88, in turn, associates with the IRAK1/TRAF6 complex. TRAF6 activates TAK1, leading to IKK complex activation. The IKK complex phosphorylates IκB, marking it for degradation and allowing NF-κB nuclear translocation. Alternatively, TAK1 activates the MAPK pathway, inducing AP-1 signaling. During HSV-2 infection, TLR3 undergoes endosomal localization and phosphorylation by c-Src, EGFR, and PI3K. TLR3 triggers TRIF-dependent assembly of the TBK1/IKKε/NAP1/TRAF3 complex, which activates both IRF3/IRF7 and NF-κB. TLR3 also utilizes TRAF and RIP1 to activate the TAB2/TAK1 complex, leading to AP-1 induction via MAPK and NF-κB activation via IKK/IκB pathways. These combined actions of NF-κB, IRF3/IRF7, and AP-1 induce type I interferon (IFN-I) and inflammatory cytokine expression, mediating innate immune protection. However, HSV-encoded proteins can subvert multiple downstream components of TLR signaling pathways.

Upon recognizing HSV-2, host cells can form endosomes to encapsulate the virus. Unc-93 homolog B1 (UNC-93B1), a transmembrane protein localized in the endoplasmic reticulum, subsequently transports TLR-3, 7, 8, and 9 from the endoplasmic reticulum to these endosomes [21]. In endosomes, TLR-3 is phosphorylated by tyrosine kinase c-Src, epidermal growth factor receptor (EGFR), and phosphatidylinositol 3-kinase (PI3K) to form dimers, initiating downstream signaling pathways. Although the direct interaction between HSV RNA and TLR-3 has not been fully confirmed, HSV-1/2 likely generate double-stranded RNA (dsRNA), which serves as a ligand for TLR-3 [21,22]. The activation of TLR-3 recruits TIR-domain-containing adaptor-inducing interferon-β (TRIF) and tumor necrosis factor receptor-associated factor (TRAF). This recruitment leads to the assembly of a signaling complex comprising TANK-binding kinase 1 (TBK1), inhibitor of nuclear factor kappa-B kinase epsilon (IKKε), NF-κB-activating kinase (NAK)-associated protein 1 (NAP1), and TRAF3, which activates interferon regulatory factors (IRF) 3, 7, and NF-κB [23]. Meanwhile, TRAF recruits TAB2 and TAK1, leading to the activation of both NF-κB and AP-1. Eventually, NF-κB, AP-1, IRF3, and IRF7 enter the nucleus, stimulating the release of various cytokines such as IFN-β, TNF-α, and IL-6 to exert antiviral effects (Figure 1).

**Figure 2:**
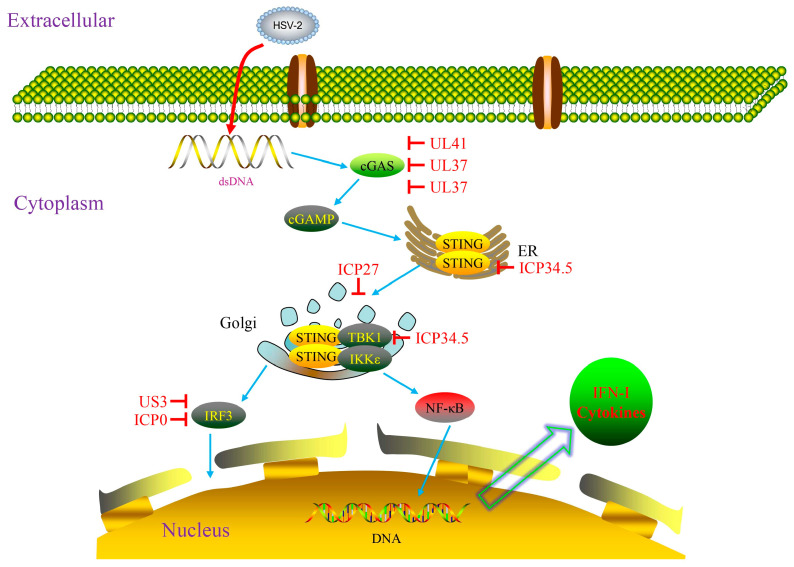
The cGAS-STING signaling pathway in HSV-2 innate immunity. Cytosolic cGAS recognizes HSV-2 dsDNA, initiating the host immune response. Upon dsDNA binding, cGAS synthesizes cGAMP. This second messenger activates the endoplasmic reticulum (ER) protein STING, triggering its translocation from the ER to the Golgi apparatus. Subsequently, STING recruits TBK1/IKKε. TBK1 phosphorylates IRF3, promoting IFN-I production, while IKKε mediates the nuclear translocation of NF-κB. Additionally, HSV-encoded proteins can target key steps downstream of the cGAS-STING signaling axis for immune evasion.

In recent years, the regulatory role of the cGAS-STING signaling pathway in the innate immune response against HSV-2 has attracted significant attention. A 2023 report demonstrated that specific genetic variants in the IKKε gene can lead to functional impairment of the cGAS-STING pathway, rendering patients susceptible to recurrent HSV-2 meningitis. The underlying mechanism involves impaired STING phosphorylation, thereby reducing IFN-β production. These findings confirm that IKKε plays an essential and non-redundant protective role within this pathway [24]. Furthermore, a 2025 review elaborated that the cGAS-STING pathway engages in a complex crosstalk network with other immune signaling pathways, including Toll-like receptors (TLRs) and NF-κB, and HSV-2 can interfere with this pathway activation to achieve immune evasion by encoding enzymes that degrade cGAMP or directly targeting STING [25] (Figure 2).

### 2.2. IFN-Mediated Antiviral Effects Against HSV-2

Type I IFNs (IFN-α, IFN-β, IFN-ε, IFN-ω, IFN-κ) constitute pivotal components of innate antiviral immunity, primarily produced by antigen-presenting cells triggered via PRRs signaling pathways. These IFNs can induce the expression of IFN-stimulated genes (ISGs), which exert antiviral effects by inhibiting viral replication and facilitating viral mRNA degradation. The antiviral activity of type I IFNs against HSV-2 was initially reported by Lopez et al. in 1975 [26], and in 2007, Svensson et al. demonstrated definitively their critical protective role in suppressing HSV-2 replication and enhancing host survival using an IFN-α/β receptor knockout mouse model [27]. Local administration of recombinant IFN-α1 or plasmid DNA encoding IFN-α1 to mice significantly restricts HSV-2 infection in both the vaginal tract and the cornea [28]. Furthermore, clinical studies have confirmed that topically applied interferon-α accelerates the cessation of viral shedding and reduces the recurrence rate in patients suffering from recurrent genital HSV-2 infection [29].

IFN-γ, the sole well-established member of the type II interferon family, is predominantly produced by activated CD4+ Th1 cells, CD8+ cytotoxic T lymphocytes (CTLs), and NK cells. Evidence indicates that ovalbumin (OVA)-specific CD8+ T cells derived from OT-I transgenic mice can effectively clear the HSV-2 tk- OVA strain (a thymidine kinase-deficient HSV-2 strain expressing OVA) from the genital tract epithelium of recipient mice; however, this clearance effect is suppressed upon IFN-γ neutralization, and CD8+ OT-I T cells deficient in IFN-γ fail to mediate this clearance ability [30]. Furthermore, IFN-γ synergizes with TNF-α to significantly inhibit HSV-2 replication in HeLa and 86HG39 cells. The antiviral efficacy of IFN-γ is dependent on augmentation by TNF-α, whereas TNF-α alone exhibits no such activity. This synergistic mechanism is associated with the activation of indoleamine 2,3-dioxygenase (IDO) [31]. Additionally, the clearance of early HSV-2 infection requires the interaction of IFN-γ with genital tract epithelial cells. This interaction potentially initiates a cascade of ISGs or upregulates molecules involved in antigen processing and presentation [32], suggesting that IFN-γ has a crucial role in protecting genital tract epithelial cells against HSV-2 infection.

Type III IFN (IFN-λ) possesses antiviral and immunomodulatory functions, and its production is regulated by viral infection and immune stimulation, mainly secreted by epithelial cells, DCs, macrophages, and neutrophils. The ISGs induced by IFN-λ are highly overlapping with those induced by type I IFNs, exerting potent local antiviral effects on barrier surfaces such as the skin and mucosal epithelia [33]. Activation of the TLR-3/retinoic acid-inducible gene I (RIG-I) signaling system in human cervical epithelial cells can trigger the production of endogenous IFN-λ, thereby enhancing the suppression of HSV-2 replication, while antibodies against IL-10Rβ (blocking the IFN-λ receptor) can weaken this inhibitory effect [34]. Mechanistic studies have shown that IFN-λ exerts its effects by inducing ISG expression, increasing PRR levels, and upregulating the expression of genes encoding Janus kinase/signal transducer and activator of transcription (JAK/STAT) signaling pathway components. Inhibition of the JAK/STAT pathway abolishes its antiviral activity [35]. A recent 2024 study revealed that IFN-λ can reduce HSV-2-induced cutaneous pathology by suppressing keratinocyte secretion of CXCL9, thereby decreasing neutrophil recruitment to the site of skin infection. Importantly, this protective effect occurs independently of a reduction in viral load and offers a novel therapeutic direction for treating viral skin infections [36].

### 2.3. Interference of HSV-Encoded Proteins with Host Innate Immunity

Comprehensively understanding the interplay between HSV and the host’s innate immune system requires looking beyond the passive detection and response to viral signatures by host molecules; equal attention must be paid to the large number of immunomodulatory molecules encoded by HSV, such as ICP0, ICP27, ICP34.5, US3, US11, UL36, UL37, UL41, and UL49. These molecules can actively shape the host immune responses through mechanisms, including suppressing activation, delaying responses, modulating response intensity, and inducing aberrant signals [14] (Figure 1 and Figure 2). As an immediate–early protein of HSV, ICP0 plays a key role in the viral lifecycle. It targets the adaptor proteins MyD88 and Mal to promote their degradation, thereby blocking the TLR2-driven NF-κB signaling pathway [37]. Concurrently, it disrupts the intranuclear distribution of ubiquitin-specific peptidase 7 (USP7), leading to the deubiquitination of TRAF6 and IKKγ and consequently inhibiting the downstream transduction of TLR signals [38]. ICP27 is a conserved multifunctional regulatory protein present in all herpesviruses, which has been shown to interact with TBK1 and stimulator of interferon genes (STING) through its RGG motif, thereby hindering IRF3 activation and type I IFN production [39]. ICP34.5, a virulence factor of HSV, can directly bind to STING, preventing its translocation from the endoplasmic reticulum to the Golgi apparatus, thus suppressing the STING-mediated antiviral signaling pathway [40]. In the early stage of infection, the envelope protein US3 inhibits the TLR-2 signaling pathway by impeding the nuclear translocation of NF-κB, resulting in reduced secretion of inflammatory cytokines such as IL-6, IL-8, and CCL2 [41]. US11, as an RNA-binding envelope protein encoded by HSV, can directly act on the C-terminal domains of RIG-I and MDA5 in both ectopic expression models and HSV-infected cell models, effectively blocking signal transduction and allowing the virus to evade innate immune surveillance [42].

UL36, the largest conserved tegument protein in herpes viruses, contains a deubiquitinase (DUB) motif at its N-terminus, also known as UL36 ubiquitin-specific protease (UL36USP). UL36USP can bind to TRAF3 and deubiquitinate it. This activity blocks IRF3 dimerization, IFN-β transcription, thereby preventing the recruitment of the downstream adapter protein TBK1. A study has found that cells infected with UL36USP-mutant virus exhibit markedly increased IFN-β production compared with those infected with wild-type HSV-1, indicating that UL36USP counteracts the RIG-I-like receptor (RLR) signaling pathway by removing polyubiquitin chains from TRAF3 [43]. UL37 (a tegument protein) acts as a deamidase to bind to RIG-I and mediates its deamidation, leading to functionally compromised RIG-I and consequent blockade of downstream antiviral immune responses [44]. Simultaneously, UL37 suppresses activation of the cytoplasmic DNA sensor cGAS, reduces synthesis of the second messenger cGAMP, and thereby blocks the STING pathway-mediated production of type I IFN [45]. The HSV tegument protein UL41 suppresses interferon signaling by degrading mRNA of the DNA sensor IFI16 [46]; VP22 (encoded by UL49) directly binds to cGAS, inhibiting its enzymatic activity and consequently reducing the production of type I IFNs and ISGs [47]. In addition, one study has revealed that the HSV-2 encoded nucleocapsid scaffold protein ICP35 possesses unique immune evasion capabilities. This protein, encoded by the UL26.5 gene and abundantly expressed during viral replication, functions as an “immunological decoy” to preferentially stimulate robust host immune responses, thereby diverting immune recognition away from critical viral antigens and facilitating the survival of the latent virus [48].

It is noteworthy that host innate immune pathways are often collaboratively hijacked by multiple HSV proteins. For example, the IFN pathway is modulated by the concerted actions of ICP0, ICP34.5, US11, and UL36. The functional network formed by these viral proteins plays a pivotal role in viral immune suppression and evasion. The dynamic game between host innate immune mechanisms and HSV immune interference strategies determines the ultimate outcome of HSV-2 infection (clearance or latency reactivation).

### 2.4. Antiviral Roles of Innate Immune Cells Against HSV-2

**Figure 3:**
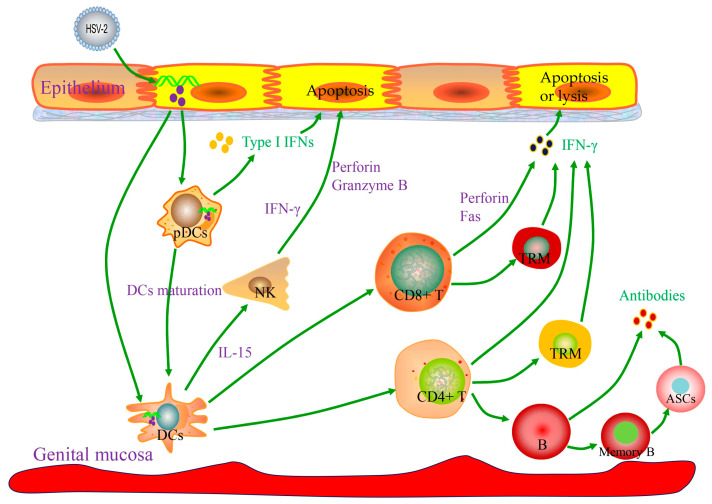
Innate immunity bridges the initial response to HSV-2 infection and activates adaptive immunity. HSV-2 infects epithelium and is detected by TLRs on epithelial cells, pDCs, DCs, and NK cells. Activated TLRs trigger the production of Type I IFNs, promoting DC maturation and IL-15 production. IL-15 facilitates NK cell survival and proliferation. NK cells release IFN-γ and directly kill infected cells via perforin and granzyme B. Ultimately, adaptive immunity mediates viral clearance. Recruited CD4+ T cells are activated by MHC class II antigen presentation on local APCs (e.g., DCs). Activated CD4+ T cells secrete IFN-γ, inducing epithelial cells to produce chemokines CXCL9 and CXCL10. These chemokines recruit cytotoxic CD8+ T cells. HSV-2-specific CD8+ T cells also release IFN-γ and kill infected cells through perforin and Fas-mediated pathways. Furthermore, CD4+ and CD8+ effector T cells can differentiate into tissue-resident memory T cells (TRMs), which are pivotal for controlling recurrence. B cells recruited to the infection site and activated by CD4+ T cells secrete antibodies. HSV-2-specific memory B cells persist long-term in peripheral lymphoid tissues and at mucosal sites. Upon viral reactivation, these memory B cells rapidly proliferate and differentiate into antibody-secreting cells (ASCs), producing high-titer HSV-2-specific IgG antibodies.

Type I IFNs can activate innate immune cells, including NK cells, DCs, and pDCs, which collectively contribute to the defense against HSV-2 infection (Figure 3).

NK cells mediate the apoptosis of virus-infected cells by releasing perforin and granzyme B and are a major source of early IFN-γ [49]. IFN-γ activates inducible nitric oxide synthase (iNOS), which catalyzes the production of nitric oxide (NO) from L-arginine, thereby inhibiting viral replication [50]. Murine studies demonstrate that NK cell deficiency or depletion increases susceptibility to HSV-2 infection and elevates viral titers in vaginal mucosa, spinal cord, and brainstem [51]. The severity of cutaneous herpes in mice with atopic dermatitis positively correlates with impaired NK cell activity [52]. Human clinical investigations have confirmed that NK cell deficiency is associated with increased susceptibility to severe HSV-2 infection [53]. Furthermore, NK cells are enriched in recurrent herpes lesions, where they interact with pDCs and CD4+ T cells; TLR-2-stimulated NK cells can directly activate HSV gD-specific CD4+ T cells, participating in the initiation of adaptive immunity [54]. Nonetheless, the role of NK cells in HSV-2 clearance remains controversial, with some studies suggesting their contribution is limited, as viral clearance primarily depends on IFN-γ-producing T cells in adaptive immunity [55].

DCs serve as a central bridge linking innate and adaptive immunity, widely distributed in the blood and peripheral tissues. They patrol between the blood and tissues, detect pathogens, and present antigens; upon receiving antigen stimulation, DCs mature and migrate to lymph nodes, where they present antigens to naïve T cells, initiating the adaptive immune response. Early studies have shown that immature DCs derived from monocytes can be effectively infected by HSV. Following virus-induced apoptosis of infected monocyte-derived DCs, uninfected bystander DCs cooperate with apoptotic DCs to cross-present HSV antigens, thereby activating HSV-specific CD8+ T lymphocytes. However, this finding necessitates validation using human tissue-derived DCs [56]. Langerhans cells (LCs), the predominant epidermal DC subset, play an important role in HSV infection. Depletion of murine skin LCs enhances HSV-1 pathogenicity, while intact LCs mediate rapid viral uptake [57]. Remarkably, human LCs retain their capacity for effective maturation, apoptosis induction, and migration to the dermal compartment after HSV infection. This difference highlights the importance of human cutaneous LCs in the immune response to HSV infection [58].

pDCs, a rare subset of DCs, are highly efficient producers of IFN-α. Concurrently, they secrete factors such as tumor necrosis factor, IL-6, CXCL10, and CCL3 to recruit and activate immune cells. Upon viral stimulation, pDCs upregulate HLA-DR, CD80, and CD86, differentiating into potent antigen-presenting cells (APCs) capable of cross-presenting exogenous antigens and activating naïve or memory CD8+ T cells [59]. Studies demonstrate that pDCs recognize HSV-2 primarily via TLR-9, exerting their antiviral function through the TLR-9/MyD88-dependent production of IFN-α, which is critically dependent on proliferation. Mouse models have confirmed an indispensable role for pDCs in innate defense against HSV-2 [60]. However, Hochrein and colleagues demonstrated that a subset of myeloid cells deficient in TLR-9 and MyD88 can still induce IFN-α production. This finding indicates that pDCs are not the exclusive source of IFN-α in HSV-2 infection and suggests that their initial recognition of the virus may occur independently of TLR-9 via unidentified pathways. Therefore, the precise underlying mechanisms warrant further investigation [61].

Innate lymphoid cells (ILCs) are categorized into ILC1, ILC2, and ILC3 subsets based on their cytokine secretion profiles and transcription factor expression (with NK cells belonging to the ILC1 subset). These subsets play critical roles in tissue remodeling, inflammation, and anti-infection responses. Research has implicated ILCs in infections caused by various pathogens, including influenza virus [62], dengue virus [63], SARS-CoV-2 [64], and *Mycobacterium tuberculosis* (Mtb) [65]. However, the role of ILCs in HSV-2 infection remains uncharacterized. Notably, Satoshi Hirose et al. demonstrated that all three ILC subsets are capable of becoming infected with HSV-1 and subsequently inhibiting its replication. They further observed that mice deficient in ILC1s or ILC3s (but not ILC2s) exhibited a marked reduction in survival rates following HSV-1 infection, suggesting that ILCs serve to restrict HSV-1 infection [66]. Given the significant structural and biological similarities between HSV-2 and HSV-1, elucidating the specific role and underlying mechanisms of ILCs in HSV-2 infection points out a direction for future study.

Recent studies on γδ T cells have garnered significant interest. A 2022 report demonstrated that the vaginal flora of diet-induced obese mice can produce L-arginine, which downregulates HIF1A expression and upregulates NKG2D receptor levels in γδ T cells through pseudo-hypoxia, thereby enhancing NO production to clear HSV-2 and significantly increase survival rates in infected mice [67].

It is crucial to emphasize that HSV infection of immune cells, such as DCs, cannot be simply attributed to the passive phagocytosis by immune cells. The key mechanism involves the active binding of viral surface glycoproteins, notably gD, to specific cellular receptors, including HVEM. This interaction signifies the initiation of the virus actively triggering the host’s defensive program [68]. Concurrently, immediately post-infection, HSV employs counter-host factors such as interferon-antagonistic proteins to suppress or reprogram the host innate immune response. This strategy synergizes with the gD-HVEM-mediated active entry mechanism, collectively forming a comprehensive viral strategy for replication and dissemination. Therefore, comprehending HSV infection and host defense mechanisms—particularly concerning the virus’s deliberate targeting of immune cells and its systematic subversion of innate immunity—necessitates situating these processes within the macroevolutionary context of host–pathogen interactions and the framework of their persistent coevolutionary dynamics. Only through this holistic perspective can the unique and universal aspects of HSV’s infection mechanisms and host defense strategies be truly understood.

## 3. Adaptive Immune Response to HSV-2 Infection

While innate immunity plays an important role in controlling HSV-2 infection, viral clearance requires the activation of adaptive immunity (Figure 3), among which T cell subset-mediated cellular immunity is the core, and the protective effect of humoral immunity is constrained by viral immune evasion mechanisms.

### 3.1. Humoral Immunity in HSV-2 Infection

HSV-2 infection induces the production of specific IgG antibodies by B cells. These antibodies interact with the virus at sites such as the vaginal epithelium and sensory nerve endings/axons, modulating viral pathogenicity [69]. In murine models, intravaginal administration of IgG antibodies isolated from HSV-immunized mice confers protection by reducing viral loads and ameliorating pathological manifestations [69]. Following immunization of rhesus macaques with an HSV-2 gC/gD/gE trivalent vaccine, the elicited plasma and mucosal neutralizing antibodies could effectively block the immune evasion mediated by gD and gE. This blocking activity, coupled with the stimulation of CD4+ T cell responses, resulted in suppressed infection progression [70]. A further study established that a trivalent subunit vaccine incorporating gC2, gD2, and gE2 induces high-titer neutralizing antibodies and confers significantly superior protection; it prevented HSV-2 infection in the dorsal root ganglia (DRG) of 97% of immunized mice, outperforming a monovalent gD2 vaccine [71]. In recent years, breakthroughs have been made in the research of neutralizing antibodies against HSV. A study reported in *Nature* in 2025 demonstrated that the nanobody Nb1_gbHSV targeting the prefusion conformation of gB can prevent the transition of gB to the post-fusion conformation by binding to a conserved epitope across the three domains, exhibiting a neutralizing activity against HSV-1/2 with an IC50 of 1.2 nM. Its small molecular size makes it particularly suitable for mucosal administration [72]. More recently, a study identified a broad-spectrum neutralizing antibody, 16F9. This antibody recognizes a conserved epitope on gB Domain I and blocks the interaction between the gH/gL complex and pre-gB. 16F9 exhibits cross-type and cross-species neutralization against multiple alpha herpes viruses, including HSV-1 and HSV-2. Importantly, 16F9 provided complete protection against herpes stromal keratitis and neonatal HSV infection in animal models [73].

However, although candidate glycoprotein vaccines can induce antibody responses in rodent models, they have failed to confer durable protection in humans [70]. The largest Herpevac vaccine trial demonstrated that the gD2 subunit antigen provided protection against genital HSV-1 infection but was ineffective against HSV-2, likely because the induced HSV-2 neutralizing antibody titers were only a quarter of those against HSV-1 [74]. In guinea pig models, the protective effect of this vaccine against genital diseases is mainly attributed to neutralizing antibodies targeting key epitopes of gD (including ID3, DL6, and MC14); broader epitope recognition was associated with stronger disease resistance [75]. In contrast, analysis of female subjects in the Herpevac trial found that compared to guinea pigs, humans produce significantly fewer neutralizing antibodies that can block key gD2 epitopes, and the antibody response to certain linear epitopes is almost completely absent in humans [74]. Therefore, quantitative assessment of the specific antibody response to key glycoprotein epitopes provides a new research direction for improving the accuracy of animal models in predicting the results of human clinical trials.

Despite the high expectations placed on highly neutralizing antibodies, antibodies induced by natural infection or experimental vaccines have failed to provide sustained protection against vaginal HSV-2 infection. Contributing factors may include HSV-2 infection or glycoprotein immunization that fails to fully induce high-level antibodies with antibody-dependent cellular cytotoxicity (ADCC) activity [69]. Additionally, the virus-encoded glycoprotein E and I complex, incorporated into both virions and infected cell membranes, functions as an Fc receptor that binds human IgG, thereby inhibiting antibody-mediated effector functions [76]. Thus, neutralizing antibodies alone are insufficient to achieve adequate immune defense against HSV-2 infection, and combination with cellular immunity strategies is required.

Moreover, the antiviral effector memory B cells situated in the vaginal mucosa appeared to play a crucial role in protecting against genital herpes. Studies revealed the presence of CD20+ B cells and antibody-secreting cells (ASCs) within inflammatory infiltrates of skin biopsy specimens from patients experiencing symptomatic HSV-2 reactivation or early healing [77]. These cells co-localized with CD4+ T cells to form immune clusters, suggesting the potential B cell and T cell crosstalk involved in immune response regulation. HSV-2-specific antibodies targeting viral surface antigens were also detected within the affected tissues, with their concentrations increasing during HSV-2 recurrence and healing, in contrast to stable levels in serum over time. In contrast, B cells, ASCs, and HSV-specific antibodies were infrequently detected in unaffected skin biopsies [77]. This suggests that HSV-2-specific memory B cells persist long-term in peripheral lymphoid tissues and at mucosal sites. Upon viral reactivation, these cells rapidly proliferate and differentiate into ASCs, secreting high-titer HSV-2-specific IgG antibodies. These antibodies block viral attachment and entry through neutralization while also enhancing the clearance of infected cells by CD8+ T cells and NK cells via ADCC. Furthermore, memory B cells induced by quadrivalent mRNA vaccine sustain robust antibody responses for up to 16 weeks [78]. These memory B cells, working synergistically with CD8+ T cell responses, effectively suppress primary, latent, and recurrent HSV-2 infections, highlighting their critical role in conferring durable immune protection.

### 3.2. Cellular Immunity in HSV-2 Infection

In infections with viruses such as HIV and HSV, T-cell-mediated immune responses, particularly antigen-specific CD4+ T cell responses, are crucial for achieving effective protection. Cytotoxic CD8+ T lymphocytes (CTLs) differentiate from naïve T cells and are activated by recognizing antigenic peptides presented by MHC class I molecules. These CTLs can trigger the apoptosis or lysis of infected cells through multiple mechanisms, including IFN-γ-mediated effects, release of perforin, or the Fas/FasL pathway. Studies have confirmed that inducing HSV-2-specific CD8+ T cell responses against a single epitope can protect mice against lethal HSV-2 challenge [79]. Human studies have also demonstrated the critical role of CD8+ T cells in defending against HSV-2 infection [75]. Following HSV-2 infection, antigen-specific CD8+ T cells differentiate into effector memory T cells (TEM) and tissue-resident memory T cells (TRM). CD8+ TRM cells establish persistent residence within the genital tract mucosa and peri-ganglionic tissues, serving as the first line of defense by rapidly detecting viral reactivation signals. These cells specifically recognize HSV-2 antigenic peptide/HLA complexes and, in response, secrete cytokines including IFN-γ and TNF-α. The resulting cytokine milieu induces the upregulation of innate antiviral genes (e.g., IFI16, TRIM22, IFITM3) in neighboring epithelial cells. Consequently, TRM cells establish a localized antiviral microenvironment that effectively restricts viral replication and dissemination [80]. Together, these findings underscore the pivotal role of the CD8+ TRM subset in mediating long-term viral control. However, CD8+ T-cell-mediated clearance is insufficient: patients with frequent herpes recurrence have persistently activated HSV-2-specific CD8+ T cells [81]. More importantly, CD8+ T-cell-deficient (CD8^−^/^−^) mice are still able to resist HSV-2 infection, indicating their inability to either prevent the establishment of latent infection or clear established infection [55]. In fact, CD4+ T cells play a pivotal role in controlling HSV-2 infection. CD4+ T-cell-depleted mice exhibit significantly delayed viral clearance and weakened protection [55]. Further demonstrating their centrality, CD4+ T cells constitute the earliest T cell population infiltrating HSV-2 infection sites, whereas CD8+ T cells are detectable only days later. Crucially, CD8+ T cells fail to migrate to vaginal epithelial infection sites in CD4+ T-cell-deficient mice. This failure is associated with the impaired production of IFN-γ by CD4+ T cells, which stimulates secretion of the chemokines CXCL9 and CXCL10, essential for recruiting CD8+ T cells [82]. Therefore, CD4+ T cells are indispensable for clearing HSV-2 from sites of acute infection, while CD8+ T cells mainly function in the late stage of the cellular immune response. Ultimately, an effective immune response relies on the synergistic interaction between both T cell subsets.

Following the establishment of HSV-2 infection, CD4+ and CD8+ effector T cells can form clusters and persist long-term within vaginal tissue, differentiating into TRM cells [83]. Evidence suggests that tissue-resident HSV-2-specific memory CD8+ T cells constitute a unique, locally maintained subset. This subset continuously monitors nerve endings to inhibit the reactivation of latent HSV-2 from nerve cells, serving as key effector cells for controlling HSV-2 recurrence [84]. In fact, the activation of tissue-resident memory CD8+ T cells depends on DCs and requires CD4+ T cell assistance during the induction phase. Their abundance has a critical impact on the reactivation of HSV-2 in both mice and humans [85]. A recent study showed that an adenovirus vaccine expressing HSV-2 RR2 (UL40) and gD proteins can induce IFN-γ+ CD4+ and CD8+ TRM cells in the dorsal root ganglia and vaginal mucosa, significantly reducing viral shedding and recurrent lesions [86,87]. Furthermore, estradiol enhances vaccine-induced CD4+ TRM cell responses in the mucosa via an IL-17-mediated pathway. Intriguingly, TRM cells alone can confer effective protection against genital HSV-2 challenge in mice [88].

## 4. Conclusions

The efficient clearance of HSV-2 infection by the host relies critically on the synergistic interaction between innate and adaptive immunity. As the first line of defense, innate immunity suppresses early viral proliferation and initiates adaptive immunity through (1) activation of TLRs and cGAS-STING signaling pathways, (2) IFN-mediated antiviral effects, and (3) engagement of immune cells such as NK cells, DCs, and γδ T cells. Conversely, HSV-2 employs immunomodulatory proteins (e.g., ICP0, ICP35) to subvert host antiviral signaling pathways, facilitating immune evasion. In adaptive immunity, neutralizing antibodies (e.g., Nb1_gbHSV, 16F9) produced by humoral immunity exert localized protection, and the development of broad-spectrum neutralizing antibodies provides new possibilities for cross-genus prevention strategies. Notably, constituting the core mechanism for viral clearance and recurrence control, CD4+ T cells drive acute infection resolution by recruiting CD8+ T cells, whereas TRM cells are pivotal in suppressing viral reactivation from latency.

Recent years have witnessed significant breakthroughs in HSV-2 intervention technologies: (1) Moderna’s mRNA-1608 vaccine (expressing gD + UL45), demonstrating a marked reduction in recurrence rates in Phase II trials, has advanced to Phase III clinical testing. (2) mRNA vaccines such as Liverna/Changchun BCHT Biotechnology’s LVRNA101 and Abogen/Arcturus’s ARCoVax, employing multi-component designs to elicit synergistic humoral and cellular immune responses, have received approval for clinical trials. (3) GSK’s novel adjuvanted vaccine has entered Phase II clinical development. Furthermore, (4) CRISPR-Cas9 technology has successfully eradicated latent virus within neuronal cells in animal models, while (5) MIT’s nanoparticle delivery system demonstrates high-efficiency penetration of the blood–nerve barrier. Collectively, these advances provide renewed hope for achieving a cure for HSV-2 infection.

As a pathogen boasting an evolutionary history spanning millennia, a large genome, and intricate structure, HSV-2 exhibits an exceptionally complex infection process and dynamic interplay with the host immune system. Future research must delve into the pathogenic mechanisms of HSV-2, its immune evasion strategies, and the synergistic regulatory networks bridging innate and adaptive immunity. Integrating cutting-edge advancements such as novel mRNA vaccines, broad-spectrum neutralizing antibodies, and gene editing technologies will be crucial for developing more effective prophylactic vaccines and therapeutic agents against HSV-2.

## Data Availability

No new data were created or analyzed in this study. Data sharing is not applicable to this article.
